# Supplementary material for: Social Interaction, Lifestyle, and Depressive Status: Mediators in the Longitudinal Relationship between Cognitive Function and Instrumental Activities of Daily Living Disability among Older Adults
Source: Int J Environ Res Public Health. 2022 Apr 1;19(7):4235. doi: 10.3390/ijerph19074235 (PMC8998450; doi:10.3390/ijerph19074235)
Supplement: Supplementary file 1 [file ijerph-19-04235-s001.zip › ijerph-1635546-supplementary.pdf]

Supplementary Table S1. Linear Regression Models and Logistic Regression Models Addressing the Mediating Effects of Social Interaction, Depression Status, and Lifestyle on the Relationship Between baseline Global Cognitive Function and IADL in 2014

	M1→X			M2→X			M3→X			Y→ X+C			Y→X+M1+M2+M3+C		
	B	95% CI	*p*	*B*	95% CI	*p*	*B*	95% CI	*p*	*OR*	95% CI	*p*	*OR*	95% CI	*p*
Global cognition	0.08	(0.05, 0.12)	< 0.001	-0.07	(-0.14, -0.01)	0.035	0.04	(0.03, 0.06)	< 0.001	0.95	(0.90, 0.99)	0.023	0.96	(0.92, 1.01)	0.112
Social interaction													0.79	(0.73, 0.85)	< 0.001
Depressive status													1.15	(1.11, 1.19)	< 0.001
Lifestyle [a]													0.83	(0.72, 0.97)	0.017
Age										1.13	(1.12, 1.15)	< 0.001	1.15	(1.13, 1.17)	< 0.001
Gender										1.60	(1.22, 2.11)	0.001	1.74	(1.30, 2.32)	< 0.001
Education										0.95	(0.91, 0.98)	0.001	0.97	(0.94, 1.01)	0.104
Marital status										1.07	(0.84, 1.35)	0.596	1.01	(0.93, 1.09)	0.891
Occupation										0.91	(0.67, 1.24)	0.538	0.98	(0.90, 1.08)	0.722
Drink at present										1.23	(0.93 1.61)	0.150	1.25	(0.94, 1.67)	0.132
Smoke at present										1.11	(0.84 1.46)	0.477	0.96	(0.71, 1.29)	0.776
BMI										1.01	(0.98, 1.04)	0.579	1.03	(1.00, 1.07)	0.070

Y, IADL disability. M1, Social interaction; M2, Depressive status; M3, Lifestyle; X, Global cognitive function; C, covariables.

[a] Lifestyle is composed of fruit, vegetable and exercise.
